# Hyposmia and Neuroimaging Signature in Community-Dwelling Older Adults

**DOI:** 10.1093/geroni/igaa057.1709

**Published:** 2020-12-16

**Authors:** Cynthia Felix, Lana Chahine, Honglei Chen, Zichun Cao, Caterina Rosano

**Affiliations:** 1 University of Pittsburgh, Pittsburgh, Pennsylvania, United States; 2 University of Pittsburgh School of Medicine, Pittsburgh, Pennsylvania, United States; 3 Michigan State University, East Lansing, Michigan, United States

## Abstract

Olfaction declines with aging, and hyposmia, or impaired sense of smell, is associated with neurodegenerative disorders including Alzheimer’s Disease (AD) and Parkinson’s Disease (PD). Neuroimaging studies of hyposmia in AD/PD patients have often examined pathology-specific brain regions. Our knowledge of neural correlates in regions that mediate olfaction in community-dwelling older adults, is limited. We quantified mean diffusivity (MD) of the gray matter (GM) using diffusion tensor imaging in a community-dwelling sample of 308 older adults (mean age: 82.9 years, 58% women, 40% black). We focused on total brain and these regions involved in olfaction- olfactory bulb, amygdala, entorhinal cortex, orbitofrontal cortex, and hippocampus. Smell was tested with a scratch-and-sniff validated odor identification test, the Brief Smell Identification Test (BSIT). Hyposmia was defined as BSIT score of ≤8, assessed about 7 years prior to neuroimaging. In our sample, 23% had hyposmia, more in in men (30%) than in women (19%). Hyposmia was not significantly associated with cardiovascular risk factors such as hypertension; diseases such as stroke; age; race; cognitive or mobility functions (all p>0.1). In linear regression models adjusted for demographics and brain atrophy (total brain gray matter volume divided by intracranial volume), hyposmia was significantly associated with higher GM MD (lower microstructural integrity) of the left orbitofrontal cortex (standardized beta: 0.142, t=2.56, p=0.011). Understanding the neural substrates involved in hyposmia in aging is an important step towards advancing research on hyposmia in non-clinic-based, community-dwelling populations.

